# Industrial-Scale Copper Wear Reduction in the Electrical Discharge Machining Through Hydrostatic Extrusion

**DOI:** 10.3390/ma19071314

**Published:** 2026-03-26

**Authors:** Jacek Skiba, Mariusz Kulczyk, Sylwia Przybysz-Gloc, Monika Skorupska, Mariusz Kobus, Kamil Nowak

**Affiliations:** 1 Institute of High Pressure Physics, Polish Academy of Sciences (Unipress), Sokołowska 29, 01-142 Warsaw, Poland; mariusz@unipress.waw.pl (M.K.); sylwia@unipress.waw.pl (S.P.-G.); monikaw@unipress.waw.pl (M.S.); 2 Gemet Elżbieta Czerwieniak, ul. Lisia 16, 05-410 Józefów, Poland; m.kobus@obrobkametalicnc.pl (M.K.); k.nowak@obrobkametalicnc.pl (K.N.)

**Keywords:** hydrostatic extrusion, grain refinement, electro discharge machining, wear analysis, application tests

## Abstract

**Highlights:**

- Cu electrodes after hydrostatic extrusion showed higher durability; wear reduced by up to 80 percent vs. commercial (Cu 99.95%-M1E).
- Hydrostatic extrusion improved machined surface quality by over 20 percent.
- Hydrostatic extrusion produced a thinner, more uniform white layer, about 30 percent thinner vs. commercial.
- Hydrostatic extrusion gave ultrafine-grained structure d_2_ = 370 nm with approximately 98.8% IACS conductivity.
- Cu after hydrostatic extrusion showed 30 percent higher hardness and 60 percent higher strength vs. commercial.

**Abstract:**

The study focused on the development and optimization of plastic deformation of pure M1E copper using an unconventional hydrostatic extrusion (HE) process aimed at improving the performance of electrodes used in electrical discharge machining (EDM). The process was designed to refine the microstructure while maintaining the high electrical conductivity required for EDM applications. Optimization of a three-stage HE process (cumulative strain ε = 2.51) resulted in the formation of an ultrafine-grained structure (d_2_ ≈ 370 nm), leading to a significant increase in mechanical strength (UTS ≈ 400 MPa) while preserving very high electrical conductivity (~99% IACS). This combination of properties is particularly important for EDM electrodes, as it allows improved wear resistance without compromising electrical performance. Due to the application-oriented nature of the study, the HE-processed copper was tested under industrial EDM conditions. Wear tests were conducted using seven electrodes of different geometries required for the production of a sample injection mold. The results demonstrated a substantial reduction in electroerosion wear of HE-processed electrodes (30–90%) compared with undeformed copper, together with up to 25% improvement in surface quality. These findings indicate that hydrostatic extrusion is an effective method for producing high performance EDM electrode materials with improved durability and machining quality.

## 1. Introduction

Electrical discharge machining (EDM) is one of the unconventional manufacturing methods that does not involve classical material removal. The history of EDM dates back to 1943, when the process was first presented [1]. In the electroerosion process, the material being machined is removed due to electrical discharges generated between the electrode and the workpiece. These discharges cause local melting and evaporation of the machined material. The only limitation of the method is that the workpiece must be electrically conductive. However, an important advantage over conventional machining methods is that the hardness of the workpiece is not a technological barrier. Thanks to this, the technique can be successfully used for the production of parts from modern engineering materials in industries such as aviation, automotive, and medicine. In these sectors, high precision and surface quality are required. The manufactured parts are usually small and often have very complex geometries, which makes conventional machining difficult or even impossible. A typical example is precision micro-holes with diameters ranging from 1 to 999 μm [2,3], which cannot be made by other methods. The most common material used to manufacture electrodes for the EDM process is pure electrolytic copper M1E. This is mainly due to its favorable physical properties, such as high electrical and thermal conductivity and good machinability [4]. Although pure copper meets the basic requirements for EDM electrode materials, it is becoming less efficient in the era of ultra-precise machining and advanced metallic materials. Therefore, efforts are continuously being made to develop improved electrode materials in order to increase process efficiency. Numerous studies on EDM machining of unconventional materials, including high-alloy and additively manufactured materials, can be found in the literature. One example is the study by Sahu et al. [5], who analyzed the electroerosion wear of Ti-5Al-2Sn alloy using Cu-W electrodes in a cooled and uncooled system. Their results showed a lower wear rate for deeply cooled electrodes. Perumal et al. [6] investigated the operational wear of electrodes in the process of drilling the hard titanium alloy Ti6Al-2Sn-4Zr-2Mo produced by 3D printing. In both cases, the authors demonstrated the superiority of copper alloys (Cu-W and CuCrZr) over conventional pure electrolytic copper. Another example is the study by Selwarian et al. [7], who investigated Si3N4-TiN intermetallic ceramic composites in EDM machining. Their results indicated the need for high-temperature-resistant electrodes made of materials such as Cu-W or graphite, due to the low conductivity of the workpiece material. Kartick et al. [8] demonstrated increased durability of CuCrZr alloy electrodes compared to pure copper in the drilling process of Haynes 25 cobalt superalloy. A significant range of literature studies also cover biomaterials such as 316 L steel. For example, Ahmed et al., who verified the effect of using different types of electrodes (Cu, brass, and Al) on the quality of the machined surface. In this case, surface quality is crucial because it directly affects peri-implant tissue growth and implant integration [9]. The latest trends and developments in the EDM industry show that the main factors hindering the development of EDM technology. These include the complexity of the physical phenomena involved, difficulties in mathematical modelling, and insufficient data on tool wear and its influence on the workpiece. A current issue is the creation of technological databases documenting EDM for a wide range of workpieces and tools. Furthermore, there is a lack of research results on EDM machining for simple steels with well-known physical, mechanical, and metallurgical properties. These steels are among the most commonly used materials in industrial production. This is confirmed, among others, by Kamenskikh et al. in their very comprehensive review [10]. In this paper, the authors present the results of research assessing the impact of hydrostatic extrusion (HE), an unconventional plastic deformation method, on improving the performance properties of pure copper used for EDM electrodes. The electrodes were applied in the machining of one of the most commonly used tool steels for injection molds: WCL/1.2343/X37CrMoV5-1.

The methodology adopted by the authors was aimed at using the cheapest solution in the form of commercially available pure copper. This avoids the use of expensive alloying elements such as W in Cu-W alloy or Cr and Zr in CuCrZr alloy, which significantly increase the costs. According to the authors, optimization of EDM electrodes using hydrostatic extrusion technology may improve existing technological solutions without requiring costly implementation. Hydrostatic extrusion is a unique plastic deformation method. Due to the triaxial stress state in the deformation zone, it enables very large strains while maintaining structural and mechanical homogeneity throughout the material volume. Research conducted at the Institute of High Pressure Physics, Polish Academy of Sciences Unipress IHPP PAS for over 45 years has confirmed the exceptional usefulness of the HE method for generating severe plastic deformation in materials. This allows materials to be given new, higher strength [11,12,13], fatigue [14,15], impact [16], tribological [17,18], and corrosion [19] properties, while improving functional properties such as machinability [20,21] and electrical conductivity [22,23]. The correctness of the concept adopted by the authors is confirmed by strictly scientific research published in 2023, presenting the impact of the HE process on the physical, mechanical, and, consequently, functional properties of pure copper M1E electrodes in the EDM process [24]. Electroerosion wear tests showed over 60% lower electrode wear after the HE process compared to commercial material. The best results obtained for finishing EDM machining characterized by low machining parameters in the form of working current, *Ir*, which do not have a destructive effect on the highly energetic microstructure obtained in the HE process. However, these earlier tests were carried out in laboratory conditions using simple cylindrical electrodes with a diameter of 10 mm. Therefore, the results cannot be directly transferred to industrial production. In real manufacturing conditions, electrodes operate under complex thermal and mechanical loads. Electrodes with complex geometries also tend to experience non-uniform wear. For this reason, the applied research conducted in real production conditions represents an important novelty of the present study. The obtained results allow direct industrial implementation, including the development of EDM electrodes made of ultrafine-grained copper produced by hydrostatic extrusion.

## 2. Materials and Methods

### 2.1. Base Materials

Pure copper 99.95% (M1E) was tested. Table 1 below presents the basic parameters of the material in its initial state.

### 2.2. Hydrostatic Extrusion

The material was deformed plastically using hydrostatic extrusion (HE) in a three-stage cumulative process from an initial diameter of 69.87 mm to a final diameter of 19.90 mm. Detailed parameters of the HE process are presented in Table 2 below.

**Figure 1:**
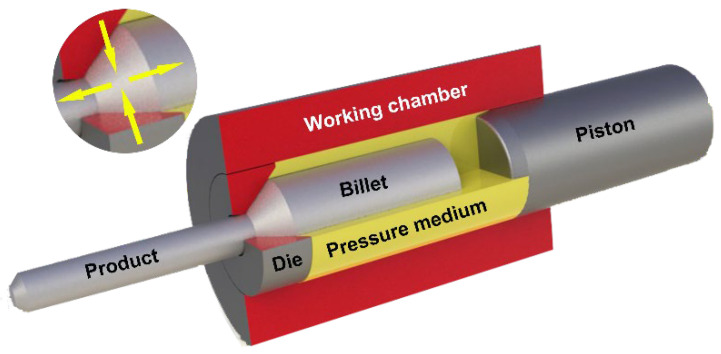
Schematic illustration of hydrostatic extrusion process.

The hydrostatic extrusion process was carried out on presses designed and manufactured at the Institute of High Pressure Physics of the Polish Academy of Sciences (IHPP PAS) with operating pressures of up to 1800 MPa, equipped with a cooling system for the extruded product, which aims to minimize the adiabatic heating effect. The HE process stations are equipped with systems for recording and archiving process parameters, including working pressure as a function of time. The figure below, Figure 1, shows a diagram of the hydrostatic extrusion process with the triaxial stress state in the deformation zone visible.

### 2.3. Mechanical Tests

The mechanical behavior of the material was investigated using a Zwick–Roell Z250universal testing machine (Zwick-Roell, Ulm, Germany), which can apply forces up to 250 kN. For each rod, five specimens with a diameter of 6 mm were extracted along the longitudinal axis and subjected to tensile testing at a constant strain rate of 0.008 s^−1^, allowing determination of yield strength (*YS*), ultimate tensile strength (*UTS*), and fracture elongation (*εf*). Additionally, microhardness measurements were performed on the cross-sectional surfaces of the extruded rods with a Zwick–Roell ZHV1-A tester (Zwick-Roell, Germany), using a 200 g load applied for 15 s.

### 2.4. Microstructure Analysis

The initial microstructure was examined using a Nikon Eclipse LV150 optical microscope (Nikon, Tokyo, Japan), whereas the microstructure following the HE process was analyzed with a JEOL 1200 EX transmission electron microscope (JOEL, Tokyo, Japan). In both cases, observations were conducted on cross-sectional slices of the extruded round rods. Quantitative evaluation of grain size was performed using the Mikrometr version 1.0 software [25]. TEM images served as the basis for the analysis: for each sample, at least 200 grains were randomly selected, and their equivalent diameter (*d*_2_) was calculated after imaging and mapping.

### 2.5. Electrical Conductivity Analysis

Electrical conductivity (%IACS) tests were performed using a SIGMATEST 2.069-Forester device (Foerster Instruments, Reutlingen, Germany). The tests were conducted on both the cross-section and longitudinal section of copper in its initial state and after the HE process.

### 2.6. Application EDM Analysis

**Figure 2:**
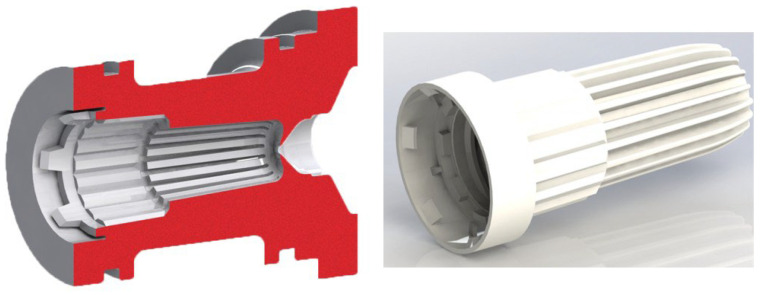
3D model of the die and self-locking nut.

In order to verify the electrodes under actual operating conditions, the authors designed a geometrically complex injection mold in the form of a self-locking nut, as shown in the photo below, Figure 2.

**Figure 3:**
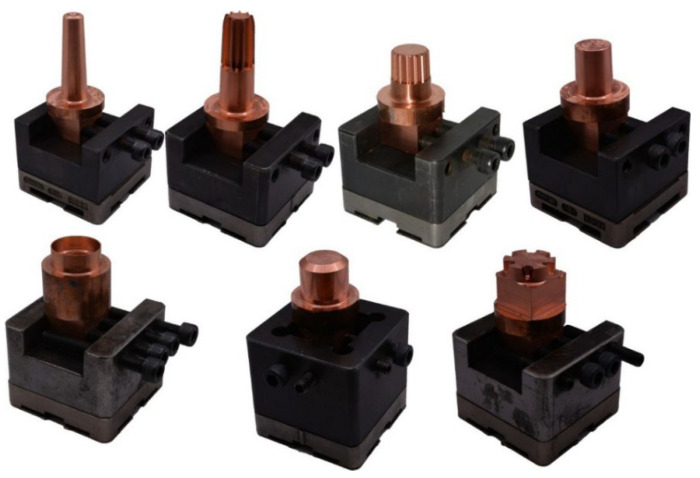
List of electrodes for the EDM process made of copper after HE.

A set of electrodes for the electroerosion process of the designed die was made from the tested material in its initial state and after the HE process. In both cases, the electrodes were made using conventional machining methods with numerically controlled machine tools. The materials were intensively cooled during the machining process, which minimized the effects of adiabatic heating. To make the die, it was necessary to make seven electrodes, each of which was designed to produce a specific geometry. Figure 3 shows a summary of the electrodes made. The EDM process was carried out on a fully automated workstation equipped with an AG FORM P 600 die-sinking EDM machine with a 3R electrode positioning system (GF Machining Solutions, Bern, Switzerland). Preliminary laboratory-scale studies conducted by the authors [24] allowed for the selection of the optimal EDM machining model. The results of preliminary laboratory-scale tests showed over 60% lower electrode wear after the HE process compared to commercial material, with the best results obtained for finishing EDM machining characterized by low machining parameters in the form of operating current, *Ir*, which do not have a destructive effect on the fine-grained, high-energy microstructure obtained in the HE process, which is susceptible to thermally induced healing and recrystallization processes.

The EDM process parameters were determined based on the VDI 3400 [26], which is mainly used in the EDM industry. Detailed EDM process data are presented in Table 3 below. The VDI 20 adopted in the EDM process corresponds to a surface roughness Ra of 1 µm.

### 2.7. Roughness Analysis

Surface roughness tests after the electroerosion process were carried out using a Hommel Tester T8000 contact profilometer from Hommelwerke with a Hommel Etamic TKU300/600 contact head (Jenoptik/Hommel-Etamic, Villingen-Schwenningen, Germany). Measurements of the selected surface topography parameter Ra were performed under the following conditions: elementary section length *lt* = 4.8 mm, head travel speed *vt* = 0.5 mm/s.

### 2.8. Electrical Discharge Wear

The wear analysis was evaluated based on the computer-aided design method using a Next Engine 3D scanner (NextEngine Inc., Santa Monica, CA, USA). Due to the highly complex shapes of EDM electrodes, electrode weight loss was evaluated for wear comparison purposes. The wear verification process was carried out using Solid Works 2021 - 3D design and modeling software.

## 3. Results and Discussion

### 3.1. Material Characterization

**Figure 4:**
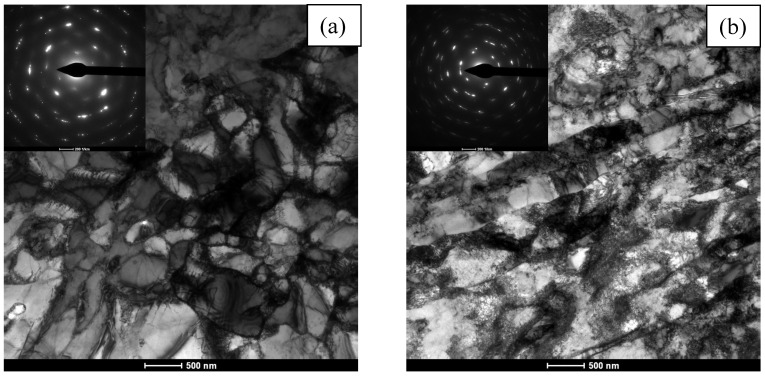
Microstructure of copper after cumulative HE with true strain ε = 2.51: (**a**) cross-section, (**b**) longitudinal section.

The material in the form of pure copper M1E intended for testing wear in the EDM process was optimized based on preliminary tests described by the authors in publication [24]. The tested copper was subjected to plastic deformation by hydrostatic extrusion (HE) in a three-stage cumulative process from an initial diameter of 69.87 mm to a final diameter of 19.90 mm. Figure 4 shows the microstructure of copper after the hydrostatic extrusion process for both the cross-section and longitudinal section of the rod, respectively, (Figure 4a and Figure 4b). In the cross-section, we observe a subgrain structure with low-angle boundaries, as evidenced by slightly blurred reflections in the SAED images. The size of the observed subgrains ranges between 200 nm and 600 nm. The average size of the observed grains/subgrains was *d*_2_ = 370 nm. In the longitudinal section, elongated, heavily defective grains are observed, arranged in bands oriented in the direction of extrusion. The thickness of the bands corresponds to the size of the subgrains observed in the cross section. In the area of oriented bands, a subgrain structure is observed locally.

**Figure 5:**
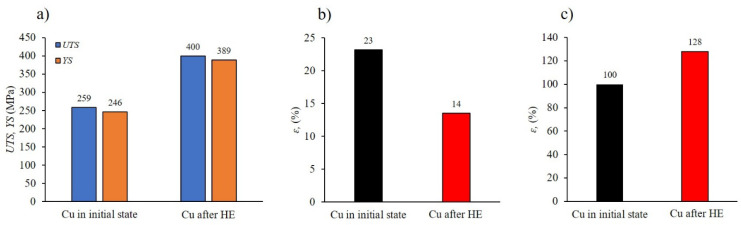
Mechanical properties of copper in initials state and after cumulative HE with true strain ε = 2.51: (**a**) strength, *UTS* and yield strength, *YS*, (**b**) elongation, *ε*, (**c**) hardness, *HV* 0.2.

The evolution of the microstructure led to a significant increase in mechanical properties, as shown in the graphs below, Figure 5.

The observed effect reflects the well-known Hall–Petch relationship, which describes the dependence of the yield strength on grain size. This phenomenon indicates that, as the grain size in a crystalline material decreases, both the yield strength and the overall mechanical strength increase. This behavior arises because grain boundaries act as barriers to the motion of dislocations; therefore, a higher density of grain boundaries impedes dislocation movement and makes plastic deformation of the material more difficult. These effects have been extensively investigated and reported in the literature, including for copper and its alloys [27,28].

The tensile strength (*UTS*) and yield strength (*YS*) values obtained increased in relation to the starting material by 54% for *UTS* and 58% for *YS*, respectively (Figure 5a). At the same time, a nearly 40% decrease in elongation at break was observed (Figure 5b), with the final elongation value of *ε* = 14% allowing for trouble-free machining of the tested copper and even facilitating it by eliminating the effect of chip ductility in the machining process. In addition, an increase in hardness of 28% was observed in the tested copper after the HE process compared to the initial material (Figure 5c). Analysis of the electrical conductivity of the tested copper did not reveal any significant changes. After three-stage HE with a true strain of 2.51, the copper had an electrical conductivity of 98.79% IACS, while the copper in its initial state had a conductivity of 100.4% IACS, which represents a decrease of approximately 1.5%. This phenomenon has been explained in detail using the example of a CuCrZr alloy after the HE process [22]. The authors’ research has shown that the characteristic grain morphology formed during the HE process—with grains elongated in the direction of extrusion—predisposes them to current flow in accordance with their orientation. Furthermore, unlike the ECAP process, the HE process ensures significantly better microstructural homogeneity, in which clearly developed grains are observed. In contrast, the structure in the ECAP process is decidedly less homogeneous. Furthermore, the uneven plastic deformation occurring in the ECAP process leads to a very intense accumulation of structural defects in the form of point, line, and volume defects, which act as an effective barrier to the flow of free electrons and phonons responsible for the transport of electrical and thermal energy.

### 3.2. TWR (Tool Wear Ratio)

There are very few reports in the literature on research into the wear of EDM electrodes manufactured using unconventional plastic deformation methods characterized by a fine-grained microstructure, and these are mainly limited to the ECAP method. An example is the study by H. Marashi et al. [29], in which the authors demonstrated a reduction in the TWR of electrodes after the ECAP process by more than 6% compared to electrodes made of undeformed material. The reduction in TWR was attributed to the higher hardness of the electrode after ECAP (1.9 GPa) than pure copper, for which the hardness was 1.7 GPa. At the same time, further research by the authors showed a deterioration in TWR with increasing deformation in subsequent stages of the ECAP process. This effect is related to the accumulation of numerous structural defects at grain boundaries, which directly affects the flow of free electrons and phonons, and consequently reduces electrical conductivity. This phenomenon has also been described in more detail for pure copper after the ECAP process by Edalati et al. [30] and Higuera-Cobos et al. [31]. In both cases, a significant decrease in electrical conductivity was observed after the ECAP process, reaching up to 20% compared to the undeformed material. Unlike the ECAP process, the HE method allows for effective microstructure refinement throughout the entire volume, while ensuring uniform deformation. As a result, the tested copper after the HE process has only a slightly lower electrical conductivity compared to the starting material, reaching approximately 1.5%, which is confirmed by the authors’ research from 2023 [24]. The advantage of the HE method in application solutions was confirmed in studies conducted by Kulczyk et al. [22], which analyzed the wear of electrodes used in the resistance welding process, made of CuCr1Zr copper alloy after the HE process and additionally strengthened by precipitation as a result of aging. The results showed a more than sixfold reduction in electrode wear after using the HE method compared to commercial electrodes. For comparison purposes, tests were also carried out on the wear of electrodes made of CuCr1Zr processed by the ECAP method. In this case, the electrodes showed only a slight improvement in properties compared to commercial electrodes, despite the clear refinement of the material’s microstructure.

**Figure 6:**
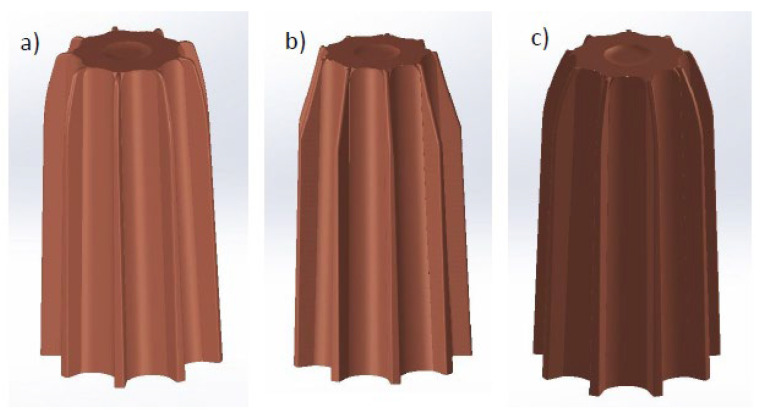
3D scans of G1 electrodes: (**a**) electrode before EDM—reference geometry, (**b**) M1E electrode in initial state after EDM, (**c**) M1E electrode after HE and after EDM.

In order to verify the electroerosion wear of M1E copper electrodes after the HE process, the EDM process was carried out in parallel on commercial material (in its initial state) and after HE. Wear analysis was evaluated based on a computer-aided design method using a Next Engine 3D scanner. The figure below, Figure 6, shows an example comparison of the geometry of the M1E electrode in its initial state and after HE in relation to the geometry of the electrode before machining.

Due to the highly complex shapes of the EDM electrodes used in the electroerosion process of the mold, the electrode mass loss was evaluated for comparison purposes. Figure 6 above shows that the wear of the M1E electrode in its initial state is significantly higher than that of the HE electrode, which is confirmed by laboratory tests on simple electrodes reported by Skiba et al. in 2023 [24]. These studies showed over 60% lower electrode wear after the HE process compared to commercial material, with the best results obtained for finishing EDM characterized by low machining parameters, including working current, *Ir*. Table 4 below presents the results of the analysis of the wear of seven prototype electrodes made of M1E in their initial state and after the HE process, as shown in Figure 3. The percentage wear rate is a measure of the relative change in the volumetric wear of a copper electrode after HE, relative to a reference value (the volumetric wear of the copper electrode in its initial state), and is expressed by the following formula:

$$W=\;\frac{W_{AR}-W_{HE}}{W_{AR}}\;\cdot 100\%$$

where

*W_AR_*—wear of the copper electrode in its initial state, and

*W_HE_*—wear of the copper electrode after the HE process.

**Table 4:** Analysis of EDM wear on prototype electrodes made of M1E, in their initial state and after hydrostatic extrusion.

Electrode Number	Electrode Volume Before EDM, (mm^3^)	Volume of the M1E AR ^1^ Electrode After EDM, (mm^3^)	M1E AR Electrode Wear, (mm^3^)	Volume of the M1E HE ^2^ Electrode After EDM, (mm^3^)	M1E HE Electrode Wear, (mm^3^)	Percentage Wear, (%)
G1	41,898.34	41,883.52	14.82	41,893.04	5.30	64.24
G2	42,772.60	42,761.17	11.43	42,766.6	6.00	47.51
G3	414.15	387.23	26.92	398.03	16.12	40.12
G4	43,801.50	43,784.98	16.52	43,792.75	8.75	47.03
G5	42,162.49	42,139.2	23.29	42,159.58	2.91	87.51
G6	5036.44	5028.19	8.25	5030.66	5.78	29.94
G7	41,038.11	41,034.64	3.47	41,036.08	2.03	41.50

^1^ M1E AR—M1E copper electrode in its initial (undeformed) state. ^2^ M1E HE—M1E copper electrode after hydrostatic extrusion process.

Due to the different geometry of the electrodes and the different drilling depths, it is not possible to make a global assessment of the wear of the tested prototype electrodes. However, in all electrodes subjected to wear testing, reduced EDM wear was observed compared to electrodes made of M1E in their initial state. This wear was reduced by between approximately 30% and over 85%.

### 3.3. Recast Layer and Heat Affected Zone (HAZ)

**Figure 7:**
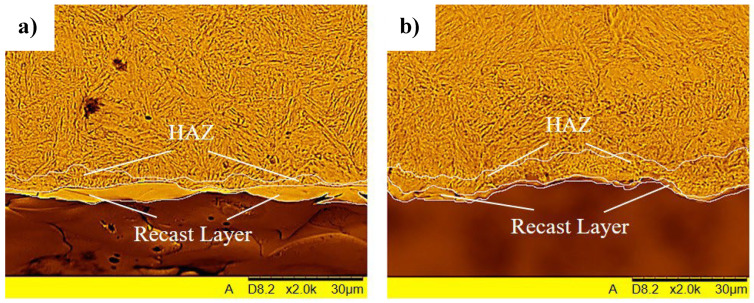
Recast layer and HAZ zone in M1E electrode after EDM process: (**a**) commercial—coarse grain (3.65 µm), (**b**) after hydrostatic extrusion—ultrafine-grained (1.83 µm).

Figure 7 shows the melted layer of material after the EDM process, known as the white layer, and the heat-affected zone (HAZ) for an example geometry of a coarse-grained electrode in its initial state and a similar fine-grained electrode after the HE process. The results clearly indicate the influence of microstructure refinement on the white layer formed during the EDM process.

This layer is significantly thinner and more uniform in the case of drilling with an electrode after the HE process than in the case of a commercial (coarse-grained) electrode. The average thickness of the layer for a commercial electrode is approximately 3.65 µm, while for an electrode after HE it is 1.83 µm, which corresponds to a 50% reduction in thickness. At the same time, in the process using the HE electrode, a slightly larger heat-affected zone (HAZ) was observed compared to the use of a commercial electrode. This effect is caused by the fragmentation of the microstructure of the HE electrode and the associated effective working surface. Materials with a fine-grained structure are characterized by a greater number of grain boundaries, which influence heat transfer and diffusion processes within the area affected by the electrical discharge. This results in more efficient heat dissipation from the locally heated area and a reduction in the volume of material that undergoes remelting. Consequently, the amount of material that solidifies again on the surface after the discharge ends in the form of a remelted layer may be reduced. Furthermore, the material’s microstructure influences the dynamics of melting and the ejection of molten metal from the erosion crater, which may also affect the final thickness of the remelted layer.

This phenomenon is also confirmed in the research of H. Marashi et al. [29], who observed similar effects for copper electrodes after the ECAP process. These studies showed that the thickness of the white layer formed during the EDM process was reduced by approximately 30% when ECAP-treated electrodes were used.

### 3.4. Surface Roughness

**Figure 8:**
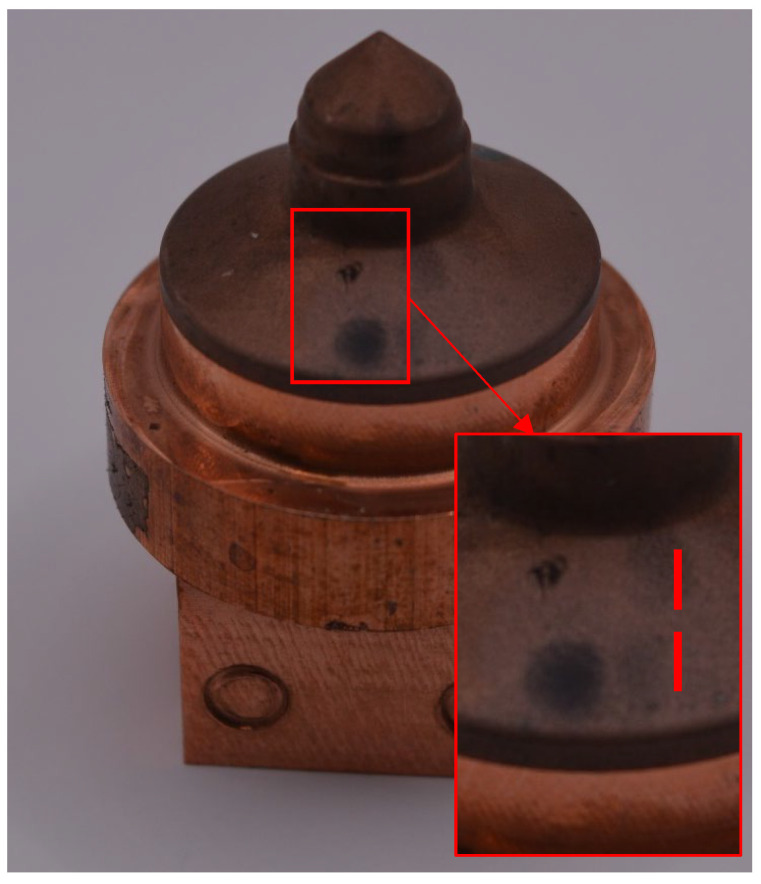
View of the G2 electrode with the marked measuring section, *lc*.

Prototype electrodes designed and manufactured based on the design of a geometrically complex injection mold in the form of a self-locking nut, Figure 2, were subjected to surface roughness analysis. Figure 8 shows an example electrode with the locations where statistical measurements of surface roughness were taken (marked with dotted lines).

In addition, Table 5 summarizes the roughness measurement results for the other prototype electrodes analyzed. The measurements were performed on electrodes made of material in its initial (undeformed) state as well as after the HE process. The results confirm the effectiveness of the developed solution, with the best results obtained for the G2 electrode (Figure 8), which is characterized by a large front surface area. For this type of electrode made of M1E after HE, the roughness was more than 20% lower than that of the electrode made of material in its initial state.

## 4. Conclusions

Pure copper is a commonly used material for manufacturing electrodes used in the EDM process. However, one of its main disadvantages is significant wear during machining, which negatively affects the accuracy of the manufactured components. In this study, hydrostatic extrusion (HE) plastic deformation was used to improve its mechanical properties and thus increase the efficiency of the EDM process. The effectiveness of this modification was then verified under actual production conditions. The analysis enabled the authors to draw the following conclusions:

1. Plastic deformation using the HE method resulted in a refined microstructure with grains elongated in the direction of extrusion, which allowed for the preservation of high electrical conductivity, which is crucial in the EDM process.
2. Copper after the HE process was also characterized by significantly higher hardness and strength than commercial material.
3. Copper electrodes after HE showed significantly higher service life compared to their commercial counterparts. Electrode wear after HE was up to 80% lower.
4. At the same time, HE electrodes exhibited a significantly thinner and more uniform white layer, which was approximately 30% thinner than that of commercial electrodes.
5. The use of HE electrodes resulted in a significant improvement in the quality of the drilled surface, reaching over 20%.

## Figures and Tables

**Table 1:** Properties of copper 99.95%-M1E in initial state (commercial material).

Material	Tensile Strength, *UTS* (MPa)	Yield Strength, *YS* (MPa)	Elongation, *ε* (%)	Hardness, *HV*_0.2_	Electrical Conductivity, IACS (*%*)
Cu 99.95%—M1E	258	246	23	101	100

**Table 2:** Basic parameters of the hydrostatic extrusion process for Cu 99.95%-M1E.

Specimen	Initial Diameter, *d*_0_ (mm)	Product Diameter, *d_f_* (mm)	True Strain, *ε* = lnR ^(a)^	Cumulative True Strain, *ε_cum_*	Adiabatic Temperature, *T* (°C)	*T/T_m_* ^(b)^	Hydrostatic Extrusion Pressure, *p_HE_* (MPa)
1CuT	69.87	39.87	1.12	1.12	119	0.29	480
1CuT-2	39.87	29.86	0.58	1.70	114	0.29	377
1CuT-3	29.86	19.90	0.81	2.51	125	0.29	460

^(a)^ R—reduction ratio = initial to final cross section. ^(b)^ Tm—melting point = 1083 °C.

**Table 3:** Summary of EDM processing parameters.

VDI	Electrode Stroke, (mm) 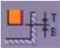	Spark Gap, (mm) 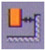	Electric Current-Max, *I_r_* (A)	Pulse Time-Max, *T* (µs)	Interval Time-Max, *P* (µm)
20	1	0.1	10.00	64.7	40.4

**Table 5:** Summary of roughness measurements, *Ra* for all types of electrodes in their initial state (AR) and after the hydrostatic extrusion (HE).

Electrode Number	Roughness, *Ra* (µm)		Percentage Difference (%)
	M1E AR ^1^	M1E HE ^2^	
G1	0.78	0.72	7.69
G2	0.97	0.74	23.85
G3	0.89	0.85	4.49
G4	0.96	0.88	8.62
G5	0.80	0.73	9.16
G6	0.88	0.83	5.32
G7	0.72	0.71	0.35

^1^ M1E AR–M1E copper electrode in its initial state (undeformed). ^2^ M1E HE–M1E copper electrode after hydrostatic extrusion process.

## Data Availability

The original contributions presented in this study are included in the article. Further inquiries can be directed to the corresponding author.

## Abbreviations

The following abbreviations are used in this manuscript:

HE	Hydrostatic Extrusion
TEM	Transmission Electron Microscopy
UTS	Ultimate Tensile Strength
YS	Yield Strength
EDM	Electrical Discharge Machining
TWR	Tool Wear Ratio
HAZ	Heat Affected Zone
E	Elongation

## References

[1] Schumacher B.M., Krampitz R., Kruth J. (2013). Historical phases of EDM development driven by the dual influence of market pull and science push. Procedia CIRP.

[2] Kumaran S.T., Ko T.J., Uthayakumar M., Adam Khan M., Niemczewska-Wójcik M. (2017). Surface texturing by dimple formation in TiAlSiZr alloy using micro-EDM. J. Aust. Ceram. Soc..

[3] Ho K.H., Newman S.T. (2003). State of the art electrical discharge machining (EDM). Int. J. Mach. Tools Manuf..

[4] Czelusniak T., Fernandes Higa C., Torres R.D., Mario De Piva J., Lohrengel A., Amorim F.L. (2019). Materials used for sinking EDM electrodes: A review. J. Braz. Soc. Mech. Sci. Eng..

[5] Sahu A.K., Mahapatra S.S. (2019). Optimization of electrical discharge machining of titanium alloy (Ti6Al4V) by grey relational analysis based firefly algorithm. Additive Manufacturing and Emerging Materials.

[6] Perumal A., Azhagurajan A., Baskaran S., Prithivirajan R., Narayansamy P. (2019). Statistical evaluation and performance analysis of EDM characteristics of hard Ti-6Al-2Sn-4Zr-2Mo alloy. Mater. Res. Express.

[7] Selvarajan L., Rajavel R., Prakash B., Mohan D.G., Gopi S. (2020). Investigation on spark EDM of Si3N4 based advanced conductive ceramic composites. Mater. Today Proc..

[8] Karthick M.P.G., Raja A.A., Chinmaya P.M., Alok C.S. (2023). Small hole fabrication through additively manufactured CuCr1Zr electrode during EDM of Haynes 25 superalloy. J. Mater. Res. Technol..

[9] Ahmed N., Abu Hurairah M., Asad Ali M., Huzaifa Raza M., Ur Rehman A., Rafaqat M. (2023). Impact analysis of electrode materials and EDM variables on surface characteristics of SS316L for biomedical applications. J. Mater. Res. Technol..

[10] Kamenskikh A.A., Muratov K.R., Shlykov E.S., Singh Sidhu S., Mahajan A., Kuznetsova Y.S., Ablyaz T.R. (2023). Recent trends and developments in the electrical discharge machining industry: A review. J. Manuf. Mater. Process..

[11] Pachla W., Kulczyk M., Swiderska-Sroda A., Lewandowska M., Garbacz H., Mazur A., Kurzydłowski K.J. Nanostructuring of metals by hydrostatic extrusion. Proceedings of the 9th International Conference on Metal Forming (EMRS).

[12] Pachla W., Kulczyk M., Sus-Ryszkowska M., Mazur A., Kurzydlowski K.J. (2008). Nanocrystalline titanium produced by hydrostatic extrusion. J. Mater. Process. Technol..

[13] Kulczyk M., Pachla W., Swiderska-Środa A., Krasilnikov N.A., Diduszko R., Mazur A., Łojkowski W., Kurzydłowski K.J. (2006). Combination of ECAP and hydrostatic extrusion for UFG microstructure generation in nickel. Solid State Phenom..

[14] Garbacz H., Motyka M., Ziaja W., Lewandowska M., Sieniawski J., Topolski K. High cycle fatigue strength of hydrostatically extruded nanocrystalline CP titanium. Proceedings of the 12th World Conference on Titanium.

[15] Garbacz H., Pakieła Z., Kurzydłowski K.J. (2010). Fatigue properties of nanocrystalline titanium. Rev. Adv. Mater. Sci..

[16] Oksiuta Z., Lewandowska M., Kurzydłowski K.J., Baluc N. (2010). Reduced activation ODS ferritic steel: Recent development in high speed hot extrusion processing. Phys. Status Solidi A.

[17] Garbacz H., Grądzka-Dahlke M., Kurzydłowski K.J. (2007). Tribological properties of nano-titanium obtained by hydrostatic extrusion. Wear.

[18] Budniak J., Lewandowska M., Pachla W., Kulczyk M., Kurzydłowski K.J. (2006). Influence of hydrostatic extrusion on properties of austenitic stainless steel. Solid State Phenom..

[19] Pisarek M., Kędzierzawski P., Janik-Czachor M., Kurzydłowski K.J. (2007). Effect of hydrostatic extrusion on resistance of 316 austenitic stainless steel to pit nucleation. Electrochem. Commun..

[20] Skiba J., Kulczyk M., Przybysz-Gloc S., Skorupska M., Niczyporuk K. (2022). Impact of severe plastic deformations obtained by hydrostatic extrusion on machinability of ultrafine-grained Ti grade 2. Sci. Rep..

[21] Skiba J., Kossakowska J., Kulczyk M., Pachla W., Przybysz S., Smalc-Koziorowska J., Przybysz M. (2020). Impact of severe plastic deformations obtained by hydrostatic extrusion on machinability of ultrafine-grained AA5083 alloy. J. Manuf. Process..

[22] Kulczyk M., Pachla W., Godek J., Smalc-Koziorowska J., Skiba J., Przybysz S., Wróblewska M., Przybysz M. (2018). Improved compromise between electrical conductivity and hardness of thermo-mechanically treated CuCrZr alloy. Mater. Sci. Eng. A.

[23] Skiba J., Kulczyk M., Przybysz-Gloc S., Skorupska M., Smalc-Koziorowska J., Kobus M., Nowak K. (2023). Thermo-mechanical treatment for reducing wear rate of CuCrZr tool electrodes during EDM. Materials.

[24] Skiba J., Kulczyk M., Przybysz-Gloc S., Skorupska M., Kobus M., Nowak K. (2023). Effect of microstructure refinement of pure copper on improving electrode performance in EDM. Sci. Rep..

[25] Wejrzanowski T., Spychalski W.L., Różniatowski K., Kurzydłowski K.J. (2008). Image based analysis of complex microstructures of engineering materials. Int. J. Appl. Math. Comput. Sci..

[26] Verein Deutscher Ingenieure (VDI) (1975). VDI 3400: Electrical Discharge Machining (EDM)—Definitions, Processes, Application.

[27] Lee J., Jeong H., Park S. (2019). Effect of extrusion ratios on microstructural evolution, textural evolution, and grain boundary character distributions of pure copper tubes during hydrostatic extrusion. Mater. Charact..

[28] Kurzydłowski K.J. (2006). Hydrostatic Extrusion as a Method of Grain Refinement in Metallic Materials. Mater. Sci. Forum.

[29] Marashi H., Jafarlou D.M., Sarahana A.A.D., Mardi N.A. (2016). Employing severe plastic deformation to processing of EDM electrodes. Precis. Eng..

[30] Edalati K., Imamura K., Kiss T., Horita Z. (2012). Equal-channel angular pressing and high-pressure torsion of pure copper: Evolution of electrical conductivity and hardness with strain. Mater. Trans..

[31] Higuera-Cobos O.F., Cabrera J.M. (2013). Mechanical, microstructural and electrical evolution of commercially pure copper processed by equal channel angular extrusion. Mater. Sci. Eng. A.

